# The importance of competition for light depends on productivity and disturbance

**DOI:** 10.1002/ece3.4403

**Published:** 2018-10-26

**Authors:** Yann Hautier, Eva Vojtech, Andy Hector

**Affiliations:** ^1^ Ecology and Biodiversity Group Department of Biology Utrecht University Utrecht The Netherlands; ^2^ Applied and Environmental Geology Department of Environmental Sciences University of Basel Basel Switzerland; ^3^ Department of Plant Sciences University of Oxford Oxford UK

**Keywords:** asymmetric competition, clipping regime, disturbance, eutrophication, light, sucrose

## Abstract

Eutrophication is a major cause of biodiversity loss. In grasslands, this appears to occur due to asymmetric competition for light following the increases in aboveground biomass production. Here, we report the results of an experiment with five grass species that tests how well‐competitive outcomes can be predicted under a factorial combination of fertilized and disturbed (frequent cutting) conditions. Under fertile conditions, our results confirm earlier success in predicting short‐term competitive outcomes based on light interception in monocultures. This effect was maintained but weakened under less fertile conditions with competition becoming more symmetric. However, under disturbed conditions, competitive outcomes could not be predicted from differences in light interception in monocultures regardless of fertility. Our results support the idea that competition in grasslands shifts from symmetric to asymmetric as fertility increases but that disturbance destroys this relationship, presumably by preventing the development of differences in canopy structure and reducing competition for light.

## 1 INTRODUCTION

Humans have greatly enhanced the rate of supply of nutrients worldwide (Galloway, Schlesinger, Levy, Michaels, & Schnoor, 1995; Tilman et al., 2001; Vitousek, Mooney, Lubchenco, & Melillo, 1997). In many types of ecosystem, this eutrophication causes loss of plant species diversity (Silvertown et al., 2006; Stevens, Dise, Mountford, & Gowing, 2004). In grasslands, size‐asymmetric competition for light has been shown to be a major mechanism of this plant diversity loss (Borer et al., 2014; DeMalach, Zaady, & Kadmon, 2017; DeMalach, Zaady, Weiner, & Kadmon, 2016; Hautier, Niklaus, & Hector, 2009). Related experiments have shown that under productive conditions the outcome of competition could be predicted from differences in light intercepting ability in monoculture (Vojtech, Loreau, Yachi, Spehn, & Hector, 2008; Vojtech, Turnbull, & Hector, 2007). However, these experiments were limited to productive conditions and were not always able to separate aboveground from belowground competition.

Using a model system of five perennial grass species commonly found in European fertile grasslands, Vojtech et al. (2007, 2008) investigated the short‐term outcome of competition for light. They performed two experiments under highly fertilized and irrigated conditions where light is assumed to be the limiting resource and competition for light to be important. In one experiment they grew all monocultures, all pairwise mixtures, and the full five‐species mixtures (see Vojtech et al., 2008). In a companion experiment (Vojtech et al., 2007) they grew one central target plant surrounded by a ring of neighbors of each of the species including itself (i.e., in all possible intraspecific and interspecific pairwise combinations). The level of incident light intercepted in monoculture, a direct measure of resource‐reduction ability, was a good predictor of short‐term competitive outcomes in pairwise mixtures (Vojtech et al., 2007, 2008). In other words, the species that reduced light resource to the lowest level in monoculture was the best competitor in pairwise mixture, consistent with Tilman's resource competition theory (Tilman, 1982, 1988). Dybzinski and Tilman (2007) found similar results over the longer‐term: light interception in monocultures of six grass species predicted competitive outcomes in pairwise mixture along a nitrogen gradient. Moreover, Vojtech et al. (2007) demonstrated that the differences in light intercepting ability conferred a disproportionate competitive advantage thereby confirming that under productive conditions competition between species for this resource is size asymmetric (Vojtech et al., 2007).

Although their experiments confirmed earlier reports of the importance of competition for light under productive conditions (Nord‐Larsen, Damaard, & Weiner, 2006; Schwinning & Weiner, 1998; Weiner, 1990), they did not test for limitation by other potential resources. In particular, they did not include measurements of belowground competition. Nevertheless, a related experiment ruled out any detectable role of belowground competition on competitive exclusion under productive conditions (Hautier et al., 2009), suggesting that belowground competition played a limited role in the predictions of competitive outcomes based on light interception abilities. However, neither experiment compares competition under fertilized conditions with that under less productive conditions.

In this paper, we report an extension of the experiment by Vojtech et al. (2008) in which we compared the predictions of competitive outcomes under productive, unproductive and disturbed conditions by applying two treatments in a fully‐factorial design. Less productive conditions were obtained by adding a carbon source in the form of sucrose and we increased disturbance of the aboveground canopy by more frequent cutting. Adding a carbon source such as sucrose or sawdust is known to stimulate soil micro‐organism immobilization of nitrogen (Killham, 1994) and competition with plants for nitrate and ammonium (Bardgett, Streeter, & Bol, 2003; Schmidt, Michelsen, & Jonasson, 1997). We aim to test if (a) under fertile and productive conditions, light interception is a good predictor of competitive outcomes and competition for light is asymmetric, (b) under unproductive conditions, light interception is still a good predictor of competitive outcomes but competition for light is symmetric, and (c) under frequent disturbance of canopy development, light interception cannot predict competitive outcomes. We show that aboveground biomass production was decreased under both unproductive and disturbed conditions thereby increasing the amount of light available in monocultures and reducing the asymmetry of competition for light. However, only when the canopy structure was frequently disturbed could the competitive outcome in pairwise mixture not be predicted from light interception levels in monoculture. Our results demonstrate that size‐asymmetric competition for light observed under fertile conditions is reduced at lower productivity and prevented by frequent disturbance of canopy development.

## 2 MATERIALS AND METHODS

### 2.1 Experimental design

The experiment reported here is part of a wider project (Vojtech et al., 2007, 2008) about light competition and partitioning in grasslands which uses a model system of five perennial grass species (Poaceae): *Alopecurus pratensis* L., *Anthoxanthum odoratum* L., *Arrhenatherum elatius* (L.) P. Beauv. ex J. Presl & C. Presl, *Festuca rubra* ssp. *commutata* Gaud. (= *Festuca nigrescens* Lam.), *Holcus lanatus* L. (Lauber & Wagner, 2001). The experiment was set up in April 2004 in the experimental garden of the Institute of Environmental Sciences, Zurich (47° 23′ N, 8° 33′ E, and 546 m height a.s.l.) and ran until June 2008 (Supporting Information Figure S1). Species were grown in 1 m^2^ plots on highly fertile soil (Garden humus; Ricoter, Aarberg, Switzerland) as five monocultures, all 10 pairwise mixtures and the single full five‐species mixtures in a fully randomized design. Each species combination was replicated five times, yielding a total of 80 plots. Species were sown at a target density of 1,000 seeds/m^2^ (corrected based on the results of prior germination trials). Plots were watered daily and weeded on a regular basis. Vojtech et al. (2007, 2008) reported the results of the first 3 years of experiment (2004–2006). During 2005 and 2006, plants were continuously fertilized to assure high nutrient amounts with a NPK fertilizer corresponding to 15 g m^−2^ year^−1^ of nitrogen in five applications of 3 g m^−2^ year^−1^ each during the growth season. Light interception and maximum canopy height were regularly monitored. Aboveground biomass was harvested in August/September in all 3 years and in June 2005 and 2006.

In 2007 we divided the plots into four subplots 50 × 50 cm. We applied two treatments in a fully‐factorial design to reduce biomass production: addition of sucrose and frequent cutting of the canopy structure. The subplots that did not receive sucrose were continuously fertilized as described above. Plots were watered daily and weeded on a regular basis. The aboveground biomass in the inner 30 × 30 cm of each subplot was harvested to a height of 3 cm in mid‐June of 2007 and 2008 and late August 2007, sorted to species, dried at 80°C and weighed. Plots received sucrose in five applications of 500 g m^−2^ year^−1^ during the growth season in 2007 and two applications of 625 g/m^2^ in 2008. The canopy structure was disturbed by increasing the number of harvests from two to four. The two additional harvests were 4 weeks before the mid‐ and late‐summer cutting typical of European meadows described above. The aboveground biomass of the additional cutting treatment in May 2008 was sorted to species, dried at 80°C and weighed. To compare the biomass production of the different treatment at the harvest of June 2008, we combined the measured biomass of the additional cutting (corrected to the inner 30 × 30 cm) to the measured biomass of the harvest of June 2008. Soil cores were collected at the end of the growth season in October 2007 and regularly during the growth season in 2008 and analyzed for nitrate and ammonium concentrations (Labor für Boden‐ und Umweltanalytik, Thun, Switzerland). Light interception was monitored at the beginning of the growing season in 2008.

### 2.2 Analysis of competition and competitive asymmetry

To investigate the importance of competition for light (cf. Vojtech et al., 2007, 2008), we related the biomass ratio of the harvest of June 2008 of each pairwise mixture to the relative difference in light interception of respective species in monoculture at the beginning of the growing season. Both the biomass ratio and the relative difference in light interception were calculated as log‐ratios of relative yield or monoculture light interception of the dominant species to the respective value of the subordinate species. We then quantified the relationship between the biomass ratio and relative differences in light interception and tested for symmetry (cf. Vojtech et al., 2007, 2008). A slope of 1 reveals symmetric competition and a slope >1 reveals asymmetric competition.

### 2.3 Statistical analysis

Biomass production was analyzed with mixed‐effects models (Pinheiro & Bates, 2000) using the lme function from the nlme library for R 3.5.0 (R Development Core Team, 2018). Treatments, number of species in the mixtures and species identity were treated as fixed effects, and species combinations, plot, and subplot were treated as random effects. As there was heterogeneity in the variance structure between species and treatment we used the varIdent() function to allow each species and each treatment to have a different variance.

Percentage of understory light availability in monocultures measured either at an early stage of vegetation growth or just before the harvest was analyzed with generalized mixed‐effects models (Gelman & Hill, 2007) using the lmer function from the lme4 library and a quasibinomial error distribution (Bates, 2005) for R 3.5.0. Treatments and species identity were treated as fixed effects, and plot was treated as random effects. In the text and graphs, we present estimates of the means from the models with their standard errors (*SEM*) and linear regression slopes with their 95% confidence intervals (95% CI).

## 3 RESULTS

### 3.1 Treatment efficacy

**Figure 1:**
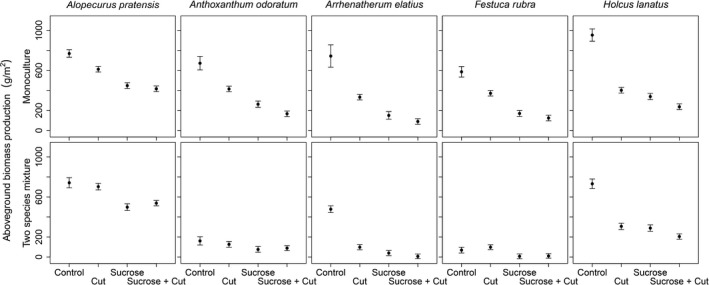
Effects of sucrose addition and frequent cutting on biomass production of five grass species in monoculture and two species mixtures. Biomass was measured at one single harvest in June during the second year of disturbance treatments addition. Points denote treatment means, and the intervals show *SEM*

In monocultures, sucrose addition and frequent cutting significantly decreased biomass production of the harvest of June 2008 (*F*_3,60_ = 155, *p* < 0.001; Figure 1, upper panels) and increased understory light availability measured before harvest (likelihood ratio test: $\chi_{3}^{2}$ = 28.7, *p* < 0.001; Supporting Information Figure S2), leading to a negative linear relationship between average light availability and biomass production (slope and 95% CI = −0.13 [−0.1 to −0.16]; Supporting Information Figure S3). Averaged over species, plants in the control subplots produced 745 ± 29 g/m^2^ (mean ± *SEM*) and transmitted 13 ± 6% of the incident light to the understory. Frequent cutting decreased productivity to 427 ± 29 g/m^2^ and increased understory light availability to 58 ± 7%. Sucrose addition decreased productivity to 274 ± 29 g/m^2^ and increased understory light availability to 65 ± 7%. The combination of frequent cutting plus sucrose addition decreased productivity to 207 ± 29 g/m^2^ and increased understory light availability to 81 ± 5%.

### 3.2 Light intercepting ability

**Figure 2:**
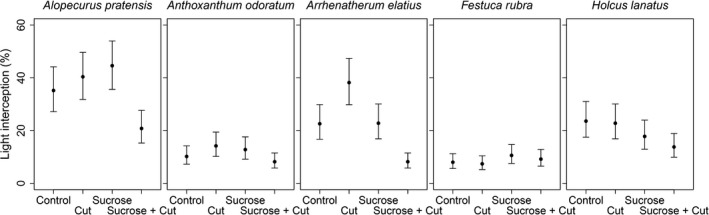
Effects of sucrose addition and frequent cutting on the percentage of incident light intercepted by five grass species in monoculture measured at the early stage of vegetation growth during the second year of treatment addition. Points denote treatment means, and the intervals show *SEM*

During the second year of our experiment, light intercepting ability in monocultures at the early stage of vegetation growth differed between species (likelihood ratio test: $\chi_{4}^{2}$ = 12.9, *p* = 0.012; Figure 2) but were not significantly different between treatments ($\chi_{3}^{2}$ = 6.4, *p* = 0.094). Averaged over treatments, *A. pratensis* intercepted 36% (30–42) of the incident light at the beginning of the growing season, *A. elatius* 23% (19–28), *H. lanatus* 20% (16–24), *A. odoratum* 11% (9–14) and *F. rubra* 9% (7–11).

### 3.3 Relationship between biomass ratio and light intercepting ability

**Figure 3:**
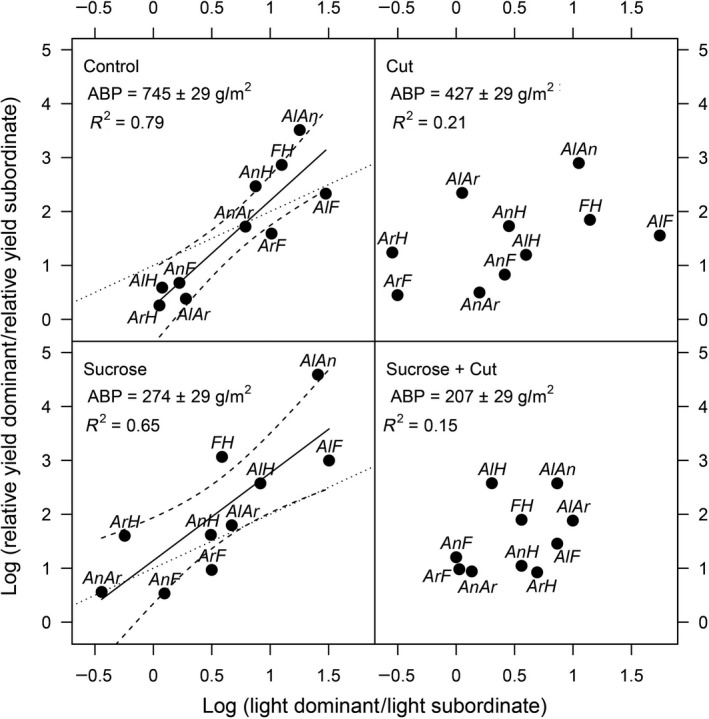
Effects of sucrose addition and frequent cutting on the relationships between the log ratio of relative biomass and the log ratio of relative difference in light interception as measured at the beginning of the growing season. Biomass ratio and relative difference in light interception were calculated as log‐ratios of relative yield or monoculture light interception of the dominant species to the respective value of the subordinate species. Results are shown as linear regression slopes and 95% CI. Dotted lines represent the expected regression line with perfect symmetry (slope of one and an intercept of zero). *Al*,* Alopecurus pratensis*;* An*,* Anthoxanthum odoratum*;* Ar*,* Arrhenatherum elatius*;* F*,* Festuca rubra*;* H*,* Holcus lanatus*; ABP, average aboveground biomass production

In the productive subplots that were not frequently cut, biomass ratio was strongly related to light intercepting ability at the early stage of vegetation growth (linear regression with 95% confidence intervals in the control subplots, Figure 3). The slope of the relationship was significantly greater than 1 (slope with 95% CI = 1.61 [1.05–2.17]) showing that higher ability to intercept incident light at early stage of vegetation growth conferred a disproportionately large competitive advantage over the growing season. This implies that under the productive conditions of our experiment competition for light was asymmetric, supporting earlier shorter term results from Vojtech et al. (2007).

After 2 years of sucrose addition, biomass ratio was still related to the ability to intercept incident light despite reduced productivity (Sucrose, Figure 3). However, the slope of the relationship was not significantly different from 1 (slope with 95% CI = 0.97 [0.53–1.42]), showing that when productivity was reduced by immobilization of nitrogen, competition was symmetric. By contrast, with frequent cutting, we found no relationship between the relative competitive effect and differences in light interception regardless of sucrose addition (Cut and Sucrose + Cut, Figure 3). This result suggests that, regardless of productivity, competitive outcomes were not driven by differences in light interception when the aboveground canopy structure of our experimental communities was disturbed.

## 4 DISCUSSION

Vojtech et al. (2007, 2008) have shown that under productive conditions, short‐term competitive outcomes could be well predicted by differences in the level of incident light intercepted in monoculture. Moreover, Vojtech et al. (2007) demonstrated that competition for light is size‐asymmetric, confirming earlier studies (Begon, 1984; Nord‐Larsen et al., 2006; Schwinning & Weiner, 1998; Weiner, 1986, 1990). After two additional years of fertilization, patterns in the control subplots of our study confirm both results: under fertilized and productive conditions light interception is an excellent predictor of competitive outcomes and competition for light is asymmetric. For example, species with comparable light intercepting ability at the early stage of vegetation growth—*A. odoratum* versus *F. rubra* or the pairwise combinations of *A. pratensis*,* A. elatius* and *H. lanatus* (Figure 2)—produced comparable biomass when grown in pairwise competition (Figure 3, control). However, when species with low light intercepting abilities—*A. odoratum* and *F. rubra*—were grown with species with higher intercepting abilities—*A. pratensis*,* A. elatius* and *H. lanatus*—they were disproportionately out‐competed in pairwise mixtures (Figure 3, control). Although we cannot identify light as the only limiting resource, a closely related experiment by Hautier et al. (2009) has shown that competition for nutrients had no detectable impact on species exclusion in similar eutrophied conditions. Therefore, our results confirm that under fertilized conditions species with a small initial advantage in light intercepting ability obtain a disproportionate share of this resource and displace poorer light competitors.

Our additional treatments significantly reduced biomass, making it possible to compare the predictions of competitive outcomes under productive condition with unproductive and disturbed conditions. Light intercepting ability measured at the early stage of vegetation growth in monocultures did not differ between treatments (Figure 2). However, understory light availability measured before harvest was significantly increased by sucrose amendment and frequent cutting (Supporting Information Figure S2), suggesting that our additional treatments successfully reduced the limitation of light over the growing season. Notably, by contrast with the productive condition, the small initial advantage of dominant species to intercept light at the early stage of vegetation growth did not lead to disproportionate competitive ability under unproductive and disturbed conditions, indicating that our treatments reduced the importance of competition for light (Figure 3).

Sucrose addition successfully reduced the amount of mineral nitrogen available to plants from 2.3 (±0.3 g/m^2^) to 0.9 (±0.3 g/m^2^) (Supporting Information Figure S4). Reduced nutrient availability decreased biomass production and increased light availability over the growing season. Competitive outcomes could still be predicted by differences in light interception at the early stage of vegetation growth despite unproductive condition but competition was more symmetric (Figure 3, Sucrose). In this case, dominant species had a competitive advantage over subordinate species but divided the contested resources in proportion of competitor sizes (Blair, 2001; Casper & Cahill, 1996; Casper & Jackson, 1997; Weiner, 1990). In other words, although the position in the canopy determined dominant and subordinate species within the community, smaller individuals were not at a disadvantage in terms of exploiting resources. There are three possible explanations. First, under less productive conditions competition for light became more symmetric. However, this seems unlikely given that light is a directionally supplied resource. Second, under less productive conditions competition was acting both above and below ground leading to a more symmetric outcome. Third, competition was now primarily for belowground resources but the competitive ability of the five species for nutrients was correlated with their light intercepting ability. It will take further experiments to test these three alternative hypotheses.

Frequent cutting also decreased biomass production and increased light availability over the growing season. Competitive outcomes could not be predicted from light intercepting ability when the canopy structure was disturbed regardless of productivity (Figure 3, Cut and Sucrose + Cut). This result shows that the competitive advantage of dominant species over subordinate species disappeared with increased canopy disturbance. This suggests that altering the structure of the canopy layer with frequent cutting prevents the importance of asymmetric competition for light and gives species equal chances to compete for the limiting resources.

Carbon amendments to eutrophied soil (Baer, Blair, Collins, & Knapp, 2004; Blumenthal, Jordan, & Russelle, 2003; Corbin & D'Antonio, 2004) and clipping regime (Lulow, 2008; Tang et al., 2009) have been used as a restoration tool to lower soil nitrogen levels and increase species diversity. Our results suggest that a possible mechanism is by directly or indirectly increasing light availability to subordinate plants, preventing initial dominance pattern from being maintained and avoiding light intercepting ability dictating the outcome of competition over the growing season.

Our short‐term study could not test for limitation by all potential resources and so an additional role of other forms of competition cannot be completely discarded. Nevertheless, altogether our findings support the idea that when ecosystems receive sufficient light plants compete primarily for limiting nutrients, a size‐symmetric process that does not lead to disproportionate competitive exclusion, while with ample nutrients plants compete primarily for limiting light, a size‐asymmetric process that lead to disproportionate competitive exclusion and plant diversity loss (Borer et al., 2014; DeMalach et al., 2016, 2017; Hautier et al., 2009). Our results demonstrate that eutrophication exacerbates the importance of asymmetric competition for light relative to initial size differences with dominant plants pre‐empting incident light to a threshold under which subordinate species are disproportionately disadvantaged. In this way, initial dominance is maintained during the whole growing season and competitive exclusion can develop very rapidly within a population and have important effects on ecosystem properties.

## CONFLICT OF INTEREST

None declared.

## AUTHORS’ CONTRIBUTIONS

YH, EV, and AH conceived and designed the experiment. YH and EV performed the experiment. YH and AH analyzed the data. YH, EV, and AH wrote the paper.

## DATA ACCESSIBILITY

Data available from the Dryad Digital Repository: https://doi.org/10.5061/dryad.59kr65r.
