# Supplementary material for: The importance of competition for light depends on productivity and disturbance
Source: Ecol Evol. 2018 Oct 26;8(22):10655–61. doi: 10.1002/ece3.4403 (PMC6262729; doi:10.1002/ece3.4403)
Supplement: Supplementary file 1 [file ECE3-8-10655-s001.docx]

**Supplementary Material for: The importance of competition for light depends on productivity and disturbance**

Yann Hautier^1^, Eva Vojtech^2^ & Andy Hector^3^

1. Ecology and Biodiversity Group, Department of Biology, Utrecht University, Padualaan 8, 3584 CH Utrecht, Netherlands.

2. Applied and Environmental Geology, Department of Environmental Sciences, University of Basel, Bernoullistrasse 32, 4056 Basel, Switzerland.

3. Department of Plant Sciences, University of Oxford, South Parks Road, Oxford, OX1 3RB, UK.

*Corresponding author:* Yann Hautier, [y.hautier@uu.nl](mailto:y.hautier@uu.nl)


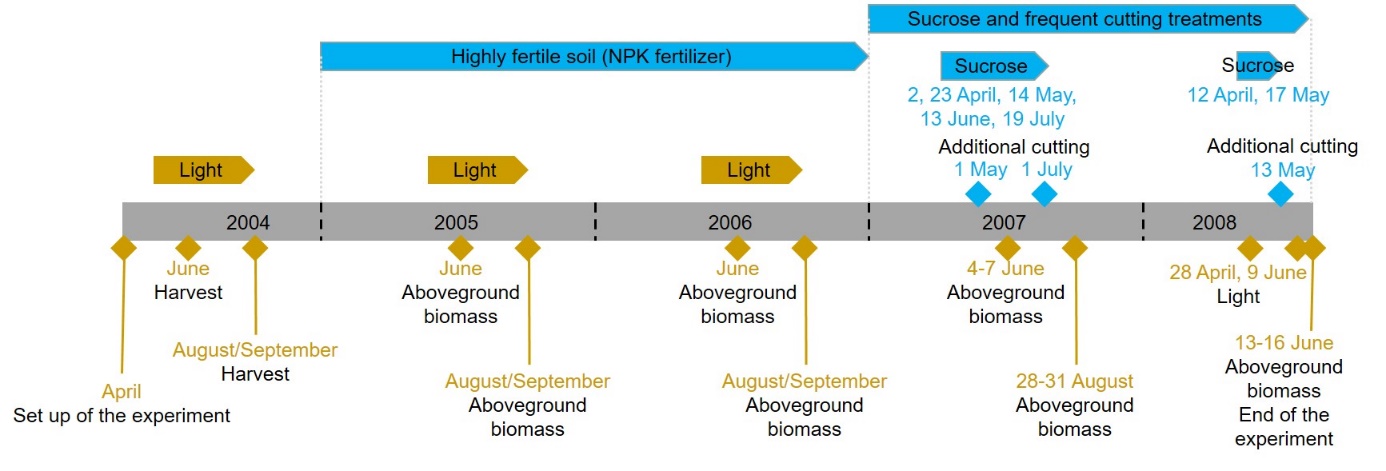


**Figure S1.** Timeline indicating when the measurements were done and the treatments applied. Data used in this paper were all collected in 2008.


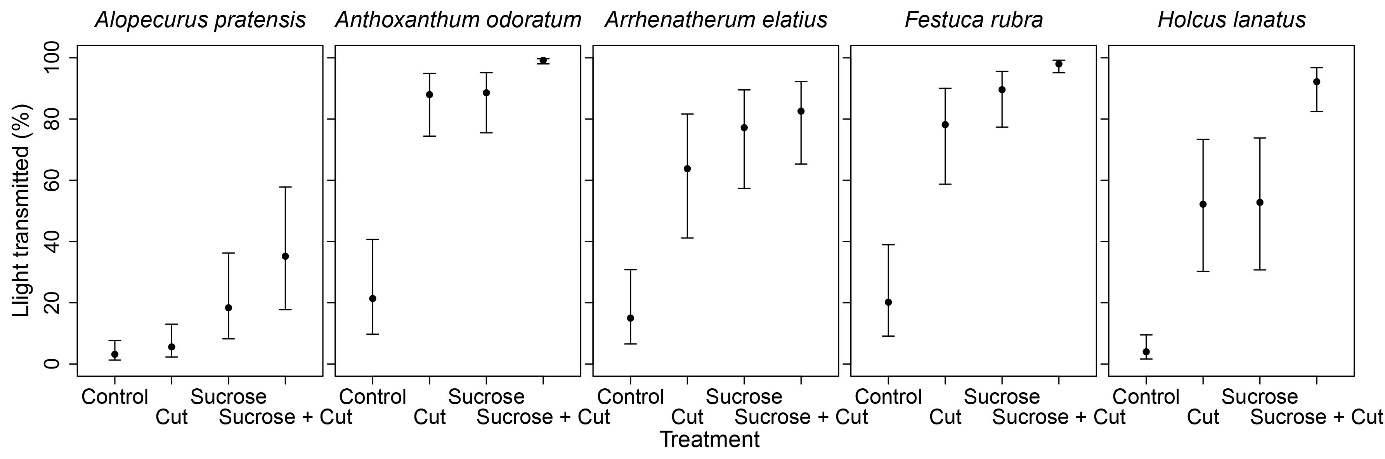


**Figure S2.** Effects of sucrose addition and frequent cutting on the percentage of incident light transmitted by five grass species in monoculture measured just before the harvest of June during the second year of treatment addition. Points denote treatment means, and the intervals show s.e.m.


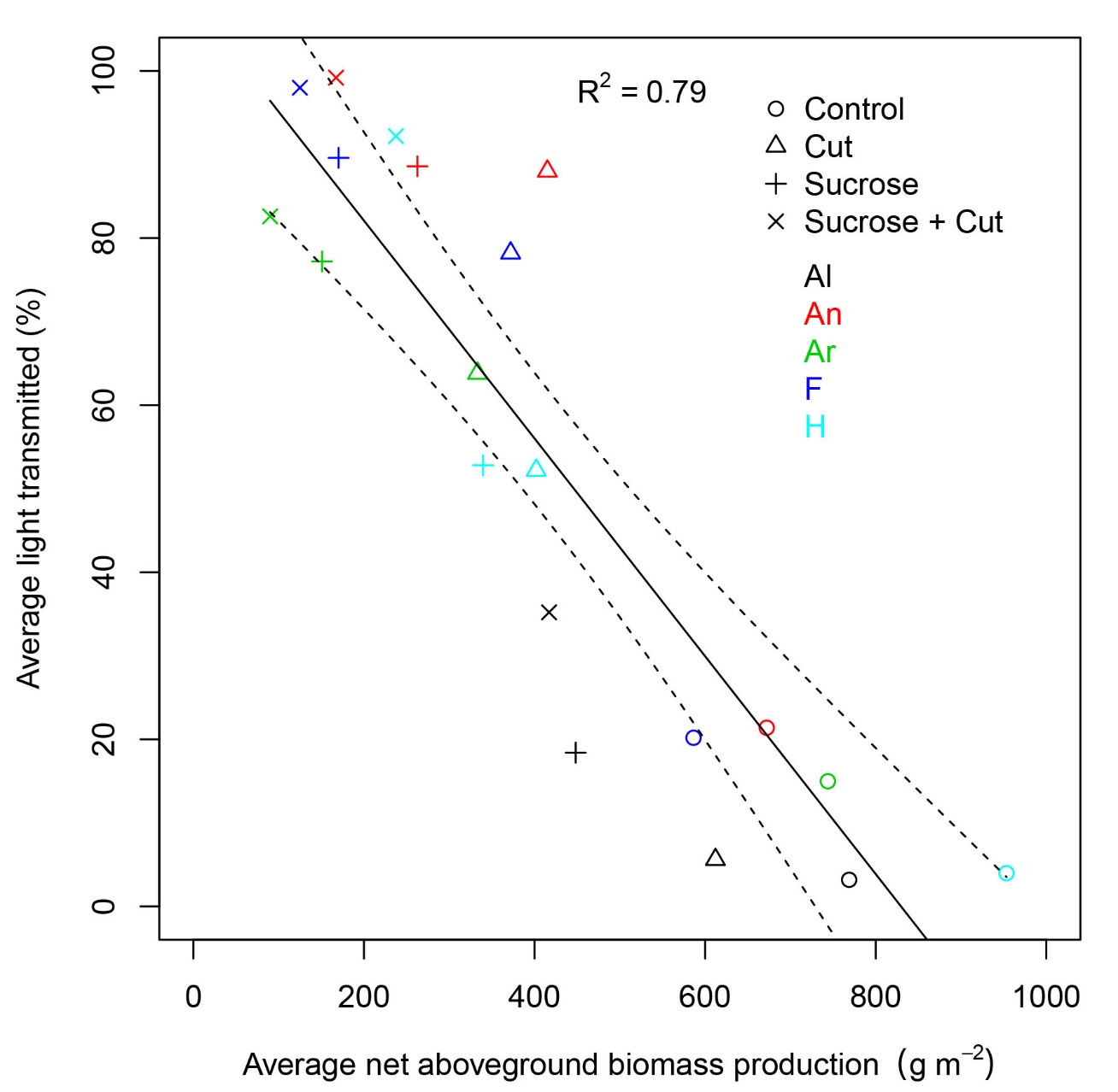


**Figure S3.** Relationship between the average aboveground biomass production and average light interception in monoculture measured before harvest. Results are shown as linear regression slopes and 95% CI. *Al* = *Alopecurus pratensis, An* = *Anthoxanthum odoratum, Ar* = *Arrhenatherum elatius, F* = *Festuca rubra* and *H* = *Holcus lanatus*. ABP = average aboveground biomass production.


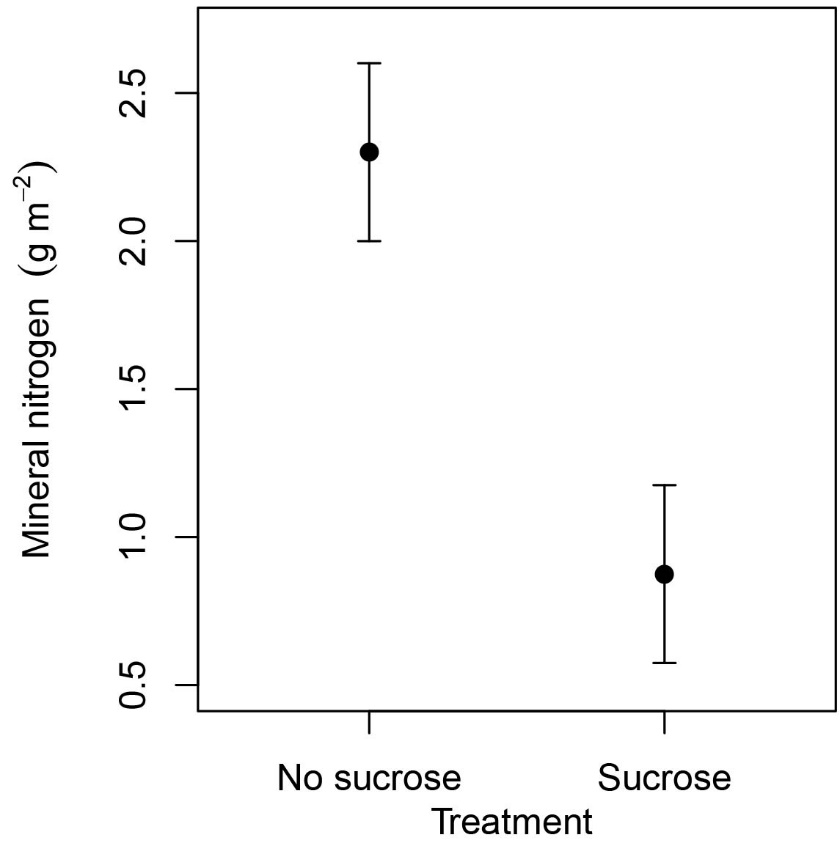


**Figure S4.** Effect of sucrose addition on the availability of mineral nitrogen. Points denote treatment means, and the intervals show s.e.m.
